# EORTC Early Clinical Studies Group early phase II trial of S-1 in patients with advanced or metastatic colorectal cancer

**DOI:** 10.1038/sj.bjc.6600781

**Published:** 2003-03-04

**Authors:** J Van den Brande, P Schöffski, J H M Schellens, A D Roth, F Duffaud, K Weigang-Köhler, F Reinke, J Wanders, R F de Boer, J B Vermorken, P Fumoleau

**Affiliations:** 1 Department of Medical Oncology, University Hospital Antwerp, Wilrijkstraat 10, 2650 Edegem, Belgium; 2 Department of Haematology and Oncology, Hannover Medical School, Carl-Neuberg Str. 1, Hannover D – 30625, Germany; 3 Antoni van Leeuwenhoekziekenhuis/The Netherlands Cancer Institute, Plesmanlaan 121, Amsterdam 1066 CX, Netherlands; 4 Department of Surgery, Geneva University Hospital, 24 Rue Micheli-du-Crest, Geneva 14 CH-1211, Switzerland; 5 Medical Oncology Unit, Centre Hospitalier la Timone, Bld J Moulin, Marseille Cedex 5 13385, France; 6 Klinikum Nürnberg, Prof. Earnst-Nathan-Str. 1, 90419; Nürnberg, Germany; 7 NDDO Oncology, Amstelveenseweg 641, Amsterdam 1081 JD, Netherlands; 8 Department of Medical Oncology, Centre René Gauducheau, Site Hospitalier Nord – Bd Jacques Monod, Saint Herblain Fn 44805, France

**Keywords:** colorectal cancer, fluoropyrimidines, oral, phase II, S-1

## Abstract

Cancer of the colon and rectum is one of the most frequent malignancies both in the US and Europe. Standard palliative therapy is based on 5-fluorouracil/folinic acid combinations, with or without oxaliplatin or irinotecan, given intravenously. Oral medication has the advantage of greater patient convenience and acceptance and potential cost savings. S-1 is a new oral fluorinated pyrimidine derivative. In a nonrandomised phase II study, patients with advanced/metastatic colorectal cancer were treated with S-1 at 40 mg m^−2^ b.i.d. for 28 consecutive days, repeated every 5 weeks, but by amendment the dose was reduced to 35 mg m^−2^ during the study because of a higher than expected number of severe adverse drug reactions. In total 47 patients with colorectal cancer were included. In the 37 evaluable patients there were nine partial responses (24%), 17 stable diseases (46%) and 11 patients had progressive disease (30%). Diarrhoea occurred frequently and was often severe: in the 40 and 35 mg m^−2^ group, respectively, 38 and 35% of the patients experienced grade 3–4 diarrhoea. The other toxicities were limited and manageable. S-1 is active in advanced colorectal cancer, but in order to establish a safer dose the drug should be subject to further investigations.

Cancer of the colon and rectum is one of the most frequent malignancies both in the US and Europe (Greenlee *et al*, 2001). In the US there are almost 100 000 new cases and almost 50 000 deaths each year. 5-Fluorouracil (5-FU) is one of the cytostatic agents that, as a single agent (but in combination with folinic acid), produces response rates averaging 20% (Skibber *et al*, 2000); irinotecan, raltitrexed and oxaliplatin have in some studies achieved similar response rates (Cunningham *et al*, 1995; Becouarn *et al*, 1998; Diaz-Rubio *et al*, 1998; Saltz *et al*, 2000). 5-Fluorouracil leads to a modest survival benefit when compared to approaches such as best supportive care (Nodic Gastrointestinal Tumor Adjuvant Therapy Group, 1992; Allen-Mersh *et al*, 1994; Hafstrom *et al*, 1994). Moreover, chemotherapy delays the occurrence or progression of symptoms by 6 months and improves symptoms without severe toxicity in 40% (Nordic Gastrointestinal Tumor Adjuvant Therapy Group, 1992; Glimelius, 1993; Allen-Mersh *et al*, 1994). Standard palliative therapy is based on 5-FU/folinic acid combinations, with or without irinotecan or oxaliplatin, given intravenously.

A meta-analysis has indicated that continuous-infusion 5-FU regimens are superior to bolus 5-FU administrations, both in terms of response and overall survival, although the increase in overall survival was limited (Meta-analysis Group in Cancer, 1998), and an unpublished EORTC/AIO randomised phase III study showed only a difference in progression-free survival (Schmoll *et al*, 2000). More recently, further improvements in response rates and survival were achieved using combinations of 5-FU/folinic acid with irinotecan or oxaliplatin (de Gramont *et al*, 2000; Douillard *et al*, 2000; Saltz *et al*, 2000). The treatments, however, are becoming more complicated this way, necessitating vascular access devices and portable delivery systems. Some grade 3–4 toxic effects such as diarrhoea are significantly more frequent in the combination treatment with irinotecan.

Therefore, new and better treatments and ways of delivering them are necessary. Oral medication has the advantage of greater patient convenience and acceptance and potential cost savings (DeMario and Ratain, 1998; Borner *et al*, 2002). 5-Fluorouracil itself is not suitable for oral administration because of the inability to achieve plasma concentrations of sufficient magnitude and the variability in oral bioavailability. Currently, there are several oral fluoropyrimidines in clinical practice or in advanced stages of development (Sharma *et al*, 2000). One of these agents is S-1. S-1 is a new oral fluorinated pyrimidine derivative, in which tegafur (FT) has been combined with two 5-FU modulating substances: 5-chloro-2,4-dihydroxypyridine (gimestat (Gimeracil®), CDHP), and potassium oxonate (otastat potassium (Oteracil®), Oxo), in a molar ratio of FT : CDHP : Oxo=1 : 0.4 : 1 (Figure 1

**Figure 1:**
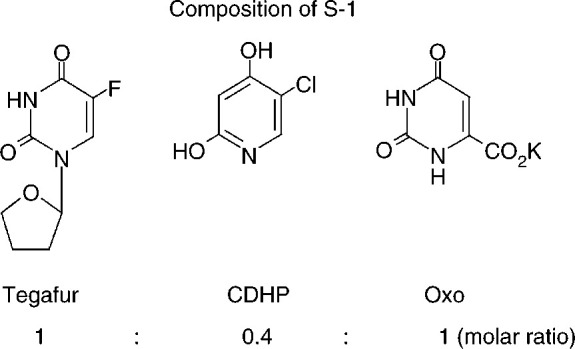
Composition of S-1.

) (Shirasaka *et al*, 1996).

The mode of action of S-1 is shown in Figure 2

**Figure 2:**
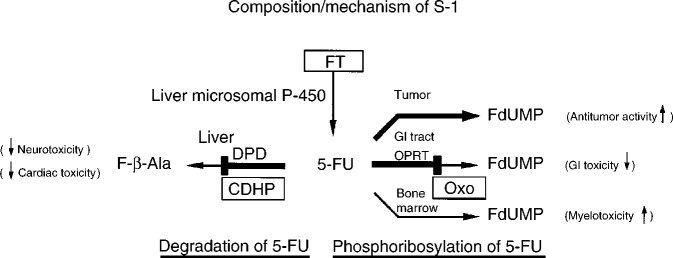
Mechanism of S-1.

. Tegafur is a prodrug of 5-FU. After oral ingestion FT is well absorbed; in the patient it is gradually converted into 5-FU, mainly in the liver and in the tumoural cells (Kimura *et al*, 1980).

CDHP inhibits the activity of dihydropyrimidine dehydrogenase (DPD), the initial and rate-limiting enzyme in the 5-FU metabolism, and thereby the degradation of 5-FU; in this respect CDHP is about 200-fold more active than uracil, which also is used in other oral combinations with FT (Tatsumi *et al*, 1987). Therefore, when 5-FU is combined with CDHP, this potentially results in the prolonged maintenance of concentrations of 5-FU, both in plasma and tumour.

Potassium oxonate prevents intestinal phosphorylation of 5-FU by inhibiting the enzyme pyrimidine phosphorybosyl transferase (Shirasaka *et al*, 1993). After oral administration, it has the potential to reduce 5-FU-induced gastrointestinal side effects (Takechi *et al*, 1997). The final mechanism of action of S-1 is exerted by 5-FU. After transformation, the cytotoxic effects of 5-FU are mediated by inhibition of the enzyme thymidylate synthase interfering with DNA synthesis, incorporation of 5-fluorouridine-5′-triphosphate (FUTP) into RNA, and incorporation of 5-fluoro-2′-deoxyuridine-5′triphosphate (FdUTP) into DNA (Peters, 1995).

S-1 has already undergone phase I and II testing. The dose-limiting toxicity was myelosuppression in a Japanese (Taguchi *et al*, 1997), and diarrhoea in a European and a North-American phase I study (Hoff *et al*, 1999; van Groeningen *et al*, 2000). The plasma pharmacokinetics of 5-FU after oral administration of S-1 were linear and almost similar to that of continuous intravenous infusion of 5-FU (Hirata *et al*, 1999). A statistically significant relation was observed between the severity of diarrhoea and pharmacokinetic parameters of 5-FU (van Groeningen *et al*, 2000). On the basis of these results, the recommended dose of S-1 in chemotherapy-naive or minimally chemotherapy-exposed patients was 40 mg m^−2^ b.i.d. for 28 consecutive days, every 5 weeks. In heavily pretreated patients, the recommended dose was 35 mg m^−2^ b.i.d. (van Groeningen *et al*, 2000).

Phase II studies showed activity of S-1 in breast cancer, head and neck cancer, colorectal, gastric cancer and nonsmall-cell lung cancer, with mild to moderate toxicity (Inuyama *et al*, 1998; Sakata *et al*, 1998; Sugimachi *et al*, 1999; Taguchi *et al*, 1998; Koizumi *et al*, 2000; Kawahara *et al*, 2001; Ohtsu *et al*, 2000). S-1 is in Japan already widely used in patients with gastric cancer and head and neck cancer. The present report is the final analysis of an early phase II study of S-1 in patients with colorectal cancer performed by the European Organization for Research and Treatment of Cancer (EORTC) Early Clinical Studies Group (ECSG).

## PATIENTS AND METHODS

### Patients

This nonrandomised phase II study was designed to determine the response rate of S-1 as first-line treatment of patients with advanced/metastatic colorectal cancer, to determine if the response rate would warrant further evaluation of S-1 in this tumour type and to further characterise the toxic effects of S-1 in this group of patients. The study was performed in accordance with the Helsinki Declaration (1964, amended in 1975 and 1983) of the World Medical Association. Eligibility criteria included histologically or cytologically verified metastatic and/or locally advanced colorectal cancer, the presence of at least one bidimensionally measurable lesion, a performance status (WHO) ⩽2, age ⩾18 years, neutrophils ⩾2000 *μ*l^−1^, platelets ⩾100 000 *μ*l^−1^, haemoglobin ⩾9 g dl^−1^, creatinine ⩽1.6 mg dl^−1^, serum bilirubin ⩽1.5 mg dl^−1^,SGPT and SGOT ⩽2× the upper limit of normal (unless related to liver metastasis), no prior chemotherapy (unless prior (neo)adjuvant chemotherapy with a treatment-free interval ⩾6 months), no poor medical risk, and written informed consent. The use of the following drugs was prohibited because of potential *in vivo* interaction with S-1: allopurinol (it diminishes S-1 activity) and phenytoin (S-1 enhances phenytoin activity).

### Treatment

The initial starting dose of S-1 was 40 mg m^−2^ b.i.d. for 28 consecutive days, repeated every 5 weeks, but by amendment the dose was reduced to 35 mg m^−2^ during the study because of a higher than expected number of severe adverse drug reactions. S-1 was administered between 7:10 am and 7:10 pm. Since Oxo is unstable in acidic conditions, patients were advised to take the capsules within 1 h after a meal. When patients were vomiting and an intact capsule was found in the stomach contents, another capsule was to be taken. Compliance of intake was monitored using patient dietary cards.

Intracycle and intercycle dose modifications and course delays for S-1 were used. When toxicity >grade 2 (exceptions: alopecia, untreated nausea/vomiting or anaemia) developed, the course was interrupted until symptoms had returned to baseline. Treatment could then be resumed at the next lower dose level, until the full 28 days were completed. If a course had to be postponed for 2 weeks because of toxicity, the next course was given at a lower dose level. For intercycle dose modifications, dose reductions were based upon toxicity in the previous course and the duration of recovery between courses. If a previous course had to be interrupted for toxicity, the patient should have been retreated in the next course at the next lower dose level, provided that this dose level did not show toxicity >grade 2. When 25 mg m^−2^ was not tolerated, the patient had to be removed from the study. Doses were not re-escalated once they had been reduced. Course delays were determined by blood counts and nonhaematological toxicities. Retreatment on day 35 took place only when the neutrophil count was 2000 *μ*l^−1^, the platelet count was ⩾100 000 *μ*l^−1^ and nonhaematological toxicities were recovered to ⩽grade 1 (exceptions: alopecia, inadequately treated nausea/vomiting, anaemia and malaise/fatigue, which should be ⩽grade 2). If these criteria were not met on day 49, the patient was taken off-study.

Ancillary treatments were given as medically indicated; the use of colony-stimulating factors was allowed when medically justified. An amendment, after inclusion of the first 35 patients, suggested that patients should start loperamide treatment at the first signs of diarrhoea: two capsules of 2 mg at the onset of diarrhoea, and thereafter one capsule of 2 mg every 2 h until 12 h after the diarrhoea had stopped. If the diarrhoea did not resolve within 24 h, S-1 had to be withheld until this was the case.

A total of two courses was planned to be given unless this was clearly not in the best interest of the patient, that is, in case of rapidly progressive disease after the first cycle or the development of acute life-threatening toxicity. If clinical response or stabilisation of disease was documented, treatment was continued until disease progression.

### Study parameters

Pretreatment evaluation included history, physical examination, WHO performance status, weight, height and tumour measurements. Laboratory procedures were complete blood count, urinalysis and biochemical tests (including serum bilirubin and creatinine); serum tumour markers were measured if applicable. All patients had an electrocardiogram (ECG), a chest X-ray and an abdominal or chest CT-scan.

Clinical studies were repeated before each course to define response and toxicity. Haematology was performed weekly and chemistries every 3 weeks. Urinalysis, chest X-ray and ECG were repeated if indicated. CT-scan of chest or abdomen was repeated every other course. In case of response this had to be confirmed ⩾4 weeks later. After an amendment (after inclusion of the first 35 patients), the abdominal or chest CT-scan had to be performed after course 1 also, in order not to miss early responses.

Toxicity was assessed according to the Common Toxicity Criteria Version 2.0 (CTC, v2.0). The responses were assessed according to the WHO criteria (World Health Organization, 1979). Patients who received a minimum of two treatment courses (i.e. 10 weeks on study) were considered evaluable for response unless rapid progression occurred in which case they were also considered evaluable. For the overall assessment of response, all parameters including both dimensionally and unidimensionally measurable lesions as well as nonmeasurable manifestations were taken into account. When progression was observed between the first and second courses, the patient was considered to be early progressive. In case patients were removed from the study earlier than 4 weeks after entry into the study, they were considered to be nonevaluable for response.

## RESULTS

Between March 1998 and August 1999, 47 patients with colorectal cancer were included in the study. In total, 46 included patients were eligible. One patient had too small lesions at enrollment. The patients were treated in six centres. After the inclusion of the first 16 patients on 40 mg m^−2^ twice daily, the protocol was amended, since some patients experienced severe diarrhoea. After implementation of this amendment, the remaining patients were included at a lower starting dose, that is, 35 mg m^−2^ twice daily. The patient and tumour characteristics are summarised in Table 1

**Table 1:** Patient and tumour characteristics in 47 treated patients

	**Treatment group**	
	**40 mg m^−2^*n*=16**	**35 mg m^−2^*n*=31**
*Age (years)*		
Median	67.5	60
Range	46–75	37–73
*WHO performance status*		
0	10	22
1	6	9
2	0	0
Median	0	0
*Sex*		
Female	6	11
Male	10	20
Prior surgery	15	29
Prior radiotherapy and/or adjuvant systemic therapy	3	7

.

At the 40 mg m^−2^ dose level 11 out of 16 patients (69%) continued treatment for a second course. Three out of 11 (27%) required a dose reduction at course 2. In total, 48 courses were given to these 16 patients. The median number of courses at this dose level was 2.5 (range 1–8).

At the 35 mg m^−2^ dose level 25 out of 31 patients (81%) continued treatment for a second course. Nine out of 25 (36%) had a dose reduction at course 2. In total, 126 courses have been given to 31 patients. The median number of courses at this dose level was 3 (range 1–24).

### Antitumour activity

Responses were initially judged by the investigators; after that, all the complete/partial responses and the long-term stable diseases were reviewed by an independent radiologist. Ten patients were considered not evaluable; that is, eight went off study early because of toxicity, one patient died early because of progressive disease, and in one patient the lesions were too small. In the remaining 37 evaluable patients there were nine partial responses (24%), 17 stable diseases (46%) and 11 patients had progressive disease (30%). Table 2

**Table 2:** Response by dose level

**WHO response criteria**	**40 mg m^−2^ group (*n*=16)**	**35 mg m^−2^ group (*n*=31)**	**All patients (*n*=47)**
Complete response	0	0	0
Partial response	2	7	9
No change	7	10	17
Progressive disease	2	9	11
(and early PD)			
Nonevaluable	5	5	10
			
*Primary site of disease*			
Colon	10	21	31
Rectum	4	5	9
Rectosigmoid	2	5	7
			
Overall response (−%)	12.5	22.6	19
(confidence interval)	(2.3–34.4)	(11.1–38.3)	(7.0–37.7)
Overall response (+%)	18.2	26.9	24
(confidence interval)	(3.3–47.0)	(13.4–44.7)	(9.1–46.3)

(−%)=response rate on all treated patients; (+%)=response rate on all evaluable patients. PD=progressive disease.

gives an overview of the responses by dose level and in relation to evaluation status. There were no remarkable differences in response rate by pretreatment characteristics.

Most responses were observed in the liver: nine out of 33 patients (27%) with liver metastases had a response in their liver. Three of out 19 patients (16%) with lung metastases had a response in their lungs.

For the evaluable patients the median time to progression in the 40 mg m^−2^ group was 138 days (44–439); the median time to progression in the 35 mg m^−2^ group was 99 days (28–1037). The median duration of response for the evaluable patients was 138 days at the 40 mg m^−2^ dose level and 141 days at the 35 mg m^−2^ dose level.

### Toxicity

At the 40 mg m^−2^ dose level four out of the 16 (25%) patients went off study prior to tumour evaluation because of toxicities, mainly gastrointestinal. At the 35 mg m^−2^ dose level only four out of 31 (13%) patients went off study prior to tumour evaluation, because of toxicities, also mainly gastrointestinal.

Diarrhoea occurred frequently and was often severe. At the 40 mg m^−2^ dose level 38% of the patients experienced grade 3–4 diarrhoea in the course of their treatment. Five patients (31%) had grade 1–2 diarrhoea. The unexpected high incidence of grade 3–4 diarrhoea led to lowering the starting dose of S-1 to 35 mg m^−2^. However, at the 35 mg m^−2^ dose level 35% of the patients still experienced grade 3–4 diarrhoea; 12 patients (39%) had grade 1–2 diarrhoea. An analysis on patient characteristics in order to explain the occurrence of diarrhoea could not find any other cause of the diarrhoea than S-1 itself. After implementation of an amendment emphasising the use of loperamide in case of diarrhoea (after inclusion of the first 35 patients), a substantial decrease in the incidence of severe diarrhoea appeared: the incidence of grade 4 diarrhoea in the 35 mg m^−2^ group dropped from 26 to 8%. Prior to the amendment 25 out of 35 treated patients experienced diarrhoea, of which only 14 were treated with loperamide. After the amendment nine out of 12 treated patients experienced diarrhoea, of which eight started loperamide treatment. So emphasising the need of the use of loperamide increased the use of loperamide and seemed to substantially decrease the incidence of grade 4 diarrhoea.

At the 40 mg m^−2^ dose level, three out of 16 patients (19%) had grade 3 nausea/vomiting. At the 35 mg m^−2^ dose level, four out of 31 patients (13%) had grade 3–4 nausea/vomiting.

Hand–foot syndrome occurred in four patients (9%), but was limited to grade 1–2.

Hyperbilirubinaemia occurred often; the incidence dropped from 75% of all courses to 38% of all courses after the starting dose had diminished from 40 to 35 mg m^−2^ b.i.d., but since hyperbilirubinaemia was assessed to be related to the treatment in only two out of 16 patients (12%) at 40 mg m^−2^ b.i.d. and in only three out of 31 patients (10%) at 35 mg m^−2^ b.i.d., there was no significant difference in treatment related hyperbilirubinaemia between the two dose levels.

Haematologic toxicity occurred frequently, but was mostly grade 1–2. Still, grade 3–4 neutropenia occurred in three patients (19%) in the 40 mg m^−2^ group, and in one patient (3%) in the 35 mg m^−2^ group. One patient used G-CSF. Only one patient experienced grade 3–4 thrombocytopenia, occurring in the 35 mg m^−2^ group. Grade 1–2 anaemia was frequent, but only in one patient a grade 3 anaemia occurred (2%).

No other drug-related serious toxicities were encountered.

## DISCUSSION

This phase II study shows that S-1 is active in patients with advanced colorectal cancer, but grade 3–4 diarrhoea is a frequent complication. Even after a dose reduction from 40 to 35 mg m^−2^, the incidence of grade 3–4 diarrhoea remained high. In a North-American phase I study the MTD of S-1 was determined to be even lower: 30 mg m^−2^ b.i.d., again with diarrhoea as the DLT (Hoff *et al*, 1999). Although the dose-limiting toxicity in an earlier European phase I study also was diarrhoea (van Groeningen *et al*, 2000), the dose-limiting toxicity in an earlier Japanese study on the contrary was myelosuppression (Taguchi *et al*, 1997). The reason for these differences in toxicity is unknown. Japanese phase II studies of S-1 in gastric, colorectal and nonsmall-cell lung cancer also did not show frequent grade 3–4 diarrhoea, but each 28-day treatment cycle was followed by a 14-day break in these studies (Sakata *et al*, 1998; Sugimachi *et al*, 1999; Kawahara *et al*, 2001). Notably, one of these studies used fixed doses of S-1 instead of body surface area adjusted doses (Sugimachi *et al*, 1999).

Oxo (potassium oxonate, otastat (Oteracil®)), which has the potential to reduce 5-FU-induced gastrointestinal side effects (Shirasaka *et al*, 1993), clearly failed to protect this patient group from diarrhoea. However, we think that it would be inappropriate at this time to give up on the principle of adding Oxo to reduce 5-FU toxicity in humans, since gastrointestinal toxicity was indeed prevented in the Japanese studies of S-1. It might therefore be worthwhile to consider other treatment regimens, leading to different pharmacokinetics, that is, a once daily administration or a lower starting dose or a shorter treatment duration (e.g. 2 weeks with 1 week rest). Another option could be to determine serum 5-FU levels after the first administration of S-1 (which might give an indication of the risk of diarrhoea) if a relation between the incidence and severity of diarrhoea and the 5-FU plasma levels could be demonstrated. The pharmaco-kinetics/pharmacodynamics data of this study will be reported separately.

The other toxicities were limited and manageable, but it should be emphasised again that out-clinic patients on this oral cytotoxic treatment should be closely monitored for the occurrence of any toxicity.

Other oral fluoropyrimidines for the treatment of colorectal cancer exist: capecitabine and UFT (uracil plus tegafur), UFT/folinic acid (Orzel), eniluracil and BOF A-2. Capecitabine and UFT/folinic acid already proved to be as effective as intravenous bolus 5-FU/folinic acid regimens (Pazdur *et al*, 1999; Hoff *et al*, 2001; Van Cutsem *et al*, 2001), achieving response rates of 18.9–24.8 and 12%, respectively, with less toxicity than with the 5-FU regimens. S-1 will have to be compared to those products in randomised studies to determine which oral fluoropyrimidine achieves the best results and the least toxicity.

Although the safe dose of S-1 has not been defined exactly in this study, it can be concluded that S-1 is active in advanced colorectal cancer. Further dose-finding studies seem to be warranted. Further analysis on baseline data is recommended in order to determine the cause of the high incidence and severity of diarrhoea in the treated Caucasian population, compared to previous Japanese data.
